# Supplementary material for: Patient-Factors Influencing the 2-Year Trajectory of Mental and Physical Health in Prostate Cancer Patients
Source: Curr Oncol. 2022 Oct 31;29(11):8244–60. doi: 10.3390/curroncol29110651 (PMC9689299; doi:10.3390/curroncol29110651)

**Table S1.** Responses to the 12-Item Short Form Survey (SF-12) by the Pros-IT patients at the time of diagnosis (n (%))

| | **n=1141** |
|---|---|
| 1. In general, would you say your health is | |
| Excellent or very good | 437 (38.3) |
| Good | 566 (49.6) |
| Fair or poor | 538 (12.1) |
| 2. Does your health *now* limit you in moderate activities such as moving a table, pushing a vacuum cleaner, bowling, or playing golf? | |
| Yes, a lot | 26 (2.3) |
| Yes, a little | 228 (20.0) |
| Not at all | 887 (77.7) |
| 3. Does your health *now* limit you in moderate activities such as climbing several flights of stairs? | |
| Yes, a lot | 28 (2.5) |
| Yes, a little | 193 (16.9) |
| Not at all | 920 (80.6) |
| *During the past 4 weeks*, have you had any of the following problems with your work or other regular daily activities as a result of your physical health? | |
| 4. Accomplished less than you would like | 182 (16.0) |
| 5. Were limited in the kind of work or other activities | 162 (14.2) |
| *During the past 4 weeks*, have you had any of the following problems with your work or other regular daily activities as a result of any emotional problems? | |
| 6. Accomplished less than you would like | 230 (20.2) |
| 7. Did work or activities less carefully than usual | 221 (19.4) |
| *8. During the past 4 weeks*, how much did pain interfere with your normal work (including work outside the home and housework) | |
| Not at all or a little bit | 1001 (87.8) |
| Moderately | 116 (10.2) |
| Quite a bit or extremely | 23 (2.0) |
| 9. How much of the time *during the past 4 weeks* have you felt calm and peaceful? | |
| All or most of the time | 688 (60.4) |
| A good bit or some of the time | 368 (32.3) |
| A little of the time or none | 84 (7.4) |
| 10. How much of the time *during the past 4 weeks* did you have a lot of energy? | |
| All or most of the time | 628 (55.2) |
| A good bit or some of the time | 436 (38.3) |
| A little of the time or none | 74 (6.5) |
| 11.How much of the time *during the past 4 weeks* have you felt down-hearted and blue? | |
| All or most of the time | 80 (7.0) |
| A good bit or some of the time | 335 (29.4) |
| A little of the time or none | 724 (63.6) |
| *12. During the past 4 weeks*, how much of the time has your physical health or emotional problems interfered with your social activities? | |
| All or most of time | 52 (4.6) |
| Some of the time | 192 (16.8) |
| A little or none of the time | 896 (89.6) |
| SF-12 PCS score, mean±SD | 51.9±7.2 |
| SF-12 MCS score, mean±SD | 49.5±9.4 |

**Table S2.** Characteristics at the time of diagnosis of the Pros-IT patients classified according to the three trajectories identified by the Mental Component Score (MCS) of the Short-Form Health Survey (SF-12)

| | Trajectory 1 (n=143) | Trajectory 2 (n=146) | Trajectory 3 (n=869) | p-value |
|---|---|---|---|---|
| Age at diagnosis, years, mean ± SD | 69.7±7.2 | 66.8±8.2 | 69.0±7.2 | 0.0079 |
| Education > lower secondary school, n (%) | 60 (42.6) | 75 (51.7) | 427 (49.8) | 0.2236 |
| BMI ≥30 kg/m$^2$, n (%) | 26 (18.4) | 23 (15.9) | 128 (15.1) | 0.7050 |
| Current smoker, n (%) | 24 (17.1) | 20 (13.8) | 122 (14.4) | 0.6588 |
| Diabetes mellitus, n (%) | 26 (18.2) | 21 (14.4) | 125 (14.5) | 0.5038 |
| 3+ moderate/severe comorbidities*, n (%) | 36 (25.2) | 29 (19.9) | 109 (12.6) | 0.0001 |
| Family history of prostate cancer, n (%) | 27 (19.2) | 33 (22.8) | 127 (14.8) | 0.0350 |
| T staging at diagnosis, n (%) | | | | 0.2181 |
| T1 | 56 (42.1) | 61 (46.2) | 440 (52.2) | |
| T2 | 61 (45.9) | 58 (43.9) | 326 (38.6) | |
| T3 or T4 | 16 (12.0) | 13 (9.9) | 78 (9.2) | |
| Gleason score at diagnosis, n (%) | | | | 0.1149 |
| ≤6 | 59 (41.6) | 64 (44.4) | 412 (47.8) | |
| 3+4 | 34 (23.9) | 28 (19.4) | 217 (25.2) | |
| 4+3 | 27 (19.0) | 26 (18.1) | 104 (12.0) | |
| ≥8 | 22 (15.5) | 26 (18.1) | 129 (15.0) | |
| PSA at diagnosis, ng/mL, median (Q1, Q3) | 7.7 (5.2, 11) | 8.2 (5.5, 11) | 6.8 (5.1, 9.7) | 0.0082 |
| D'Amico risk class, n (%) | | | | 0.2527 |
| Low | 27 (19.2) | 38 (26.4) | 238 (28.0) | |
| Intermediate | 67 (47.9) | 59 (41.0) | 368 (43.2) | |
| High | 46 (32.9) | 47 (32.6) | 245 (28.8) | |
| UCLA PCI UF, mean ± SD | 92.0±13.1 | 91.2±18.0 | 94.4±14.8 | <0.0001 |
| UCLA PCI UB, mean ± SD | 82.0±27.2 | 83.9±28.3 | 91.1±20.4 | <0.0001 |
| UCLA PCI BF, mean ± SD | 86.9±18.7 | 91.2±14.1 | 95.1±11.8 | <0.0001 |
| UCLA PCI BB, mean ± SD | 84.8±26.3 | 92.1±17.8 | 95.5±15.2 | <0.0001 |
| UCLA PCI SF, mean ± SD | 39.7±33.0 | 48.6±29.7 | 52.3±31.4 | <0.0001 |
| UCLA PCI SB, mean ± SD | 53.8±35.2 | 56.7±36.2 | 66.8±33.9 | <0.0001 |
| SF-12 PCS, mean ± SD | 49.4±9.7 | 52.6±8.8 | 52.2±6.3 | 0.0026 |
| SF-12 MCS, mean ± SD | 38.8±8.2 | 33.5±5.3 | 53.9±5.2 | <0.0001 |

**Table S3.** Characteristics at the time of diagnosis of the Pros-IT patients classified according to the two trajectories identified by the Physical Component Score (PCS) of the Short-Form Health Survey (SF-12)

| | **Trajectory 1 (n=168)** | **Trajectory 2 (n=990)** | **p-value** |
|---|---|---|---|
| Age at diagnosis, years, mean ± SD | 70.7±6.9 | 68.5±7.4 | 0.0002 |
| Education > lower secondary school, n (%) | 69 (42.1) | 493 (50.4) | 0.0495 |
| BMI ≥30 kg/m$^2$, n (%) | 42 (25.6) | 135 (14.0) | 0.0002 |
| Current smoker, n (%) | 21 (12.9) | 145 (14.9) | 0.4933 |
| Diabetes mellitus, n (%) | 39 (23.4) | 133 (13.5) | 0.0009 |
| 3+ moderate/severe comorbidities*, n (%) | 45 (27.0) | 129 (13.0) | <0.0001 |
| Family history of prostate cancer, n (%) | 25 (15.0) | 162 (16.6) | 0.6065 |
| T staging at diagnosis, n (%) | | | 0.1867 |
| T1 | 71 (43.8) | 486 (51.3) | |
| T2 | 75 (46.3) | 370 (39.1) | |
| T3 or T4 | 16 (9.9) | 91 (9.6) | |
| Gleason score at diagnosis, n (%) | | | 0.0037 |
| ≤6 | 62 (37.4) | 473 (48.2) | |
| 3+4 | 36 (21.7) | 243 (24.8) | |
| 4+3 | 32 (19.4) | 125 (12.7) | |
| ≥8 | 36 (21.7) | 141 (14.3) | |
| PSA at diagnosis, ng/mL, median (Q1, Q3) | 7.4 (5.5, 11.4) | 6.9 (5.1, 9.8) | 0.0178 |
| D'Amico risk class, n (%) | | | 0.0007 |
| Low | 25 (15.3) | 278 (28.6) | |
| Intermediate | 76 (46.3) | 418 (43.1) | |
| High | 63 (38.4) | 275 (28.3) | |
| UCLA PCI UF, mean ± SD | 90.7±17.7 | 94.2±14.5 | 0.0002 |
| UCLA PCI UB, mean ± SD | 82.0±28.1 | 90.2±21.5 | <0.0001 |
| UCLA PCI BF, mean ± SD | 89.2±17.3 | 94.3±12.5 | <0.0001 |
| UCLA PCI BB, mean ± SD | 85.6±25.7 | 95.1±15.5 | <0.0001 |
| UCLA PCI SF, mean ± SD | 32.2±29.8 | 53.2±31.0 | <0.0001 |
| UCLA PCI SB, mean ± SD | 55.5±36.0 | 65.3±34.4 | 0.0012 |
| SF-12 PCS, mean ± SD | 42.7±9.4 | 53.4±5.5 | <0.0001 |
| SF-12 MCS, mean ± SD | 45.9±10.5 | 50.0±9.4 | <0.0001 |

**Stratification by age**

- MCS

Stratifying patients by age led to identifying two variables predicting MCS trajectories: among patients younger than 70 years at diagnosis, a family history of prostate cancer (OR=2.19, 95% CI 1.18-4.05) and UF, BF and SF scores at diagnosis in the highest quartile were significantly associated with the *Recovering Group* membership (OR=0.46, 95% CI 0.24-0.89; OR=0.42, 95% CI 0.22-0.79; OR=0.53, 95% CI 0.29-0.99, respectively). Among patients 70 or older, significant predictors were having 3 or more comorbidities for both the Recovering Group and the Permanently low score Group memberships (OR=2.36, 95% CI 1.22-4.53; OR=2.25, 95% CI 1.22-4.17, respectively) and an education level higher than lower secondary school for the Permanently Low Score membership (OR=1.95, 95% CI 1.08-3.52). In addition, UF, BF and SF scores in the highest quartile at the time of diagnosis were significantly associated with the Recovering Group (OR=0.41, 95% CI 0.23-0.71; OR=0.44, 95% CI 0.25-0.79; OR=0.28, 95% CI 0.08-0.96, respectively).

- PCS

Considering patients younger than 70 at the time of diagnosis, the only characteristic significantly associated with Recovering Group membership for PCS was having undergone RT as prostate cancer treatment, as opposed to NSRP (OR=5.98, 95% CI 1.55-23.1); a borderline significance was calculated for diabetes (OR=2.73, 95% CI 0.92-8.08). Among the patients 70 or older, only UF and BF scores in the highest quartile at the time of diagnosis were significant protective factors with respect to *trajectory 2* membership (OR=0.52, 95% CI 0.27-1.00; OR=0.39, 95% CI 0.21-0.73, respectively).

**Comparison with ISTAT findings**

**Table S4.** Mean and standard deviation by age group for MCS-12 and PCS-12 in Pros-IT and ISTAT studies

| | | MCS-12 | | | | PCS-12 | | |
|---|---|---|---|---|---|---|---|---|
| | | Pros-IT | ISTAT | | | Pros-IT | ISTAT | |
| **Age (years)** | **n Pros-IT** | **Mean±SD** | **Mean±SD** | **p-value** | **n ISTAT** | **Mean±SD** | **Mean±SD** | **p-value** |
| 45-54 | 34 | 52.5 ± 3.6 | 51.2±8.5 | 0.37 | 4774 | 54.1±4.5 | 51.8±7.5 | 0.07 |
| 55-64 | 141 | 51.3 ± 5.3 | 50.5±9.5 | 0.34 | 4130 | 52.7±4.4 | 49.2±8.9 | <0.0001 |
| 65-74 | 524 | 51.5 ± 5.5 | 49.5±10.2 | <0.0001 | 3409 | 51.0±5.7 | 45.3±10.5 | <0.0001 |
| >74 | 156 | 51.1 ± 5.8 | 46.9±11.7 | <0.0001 | 2008 | 50.0±5.6 | 40.0±11.6 | <0.0001 |

**Table S5.** Quartiles divided by age groups for PCS and MCS SF-12 distributions in Pros-IT and ISTAT studies

| | 45-54 years | | 55-64 years | | 65-74 years | | 75+ years | |
|---|---|---|---|---|---|---|---|---|
| | **Pros-IT** | **ISTAT** | **Pros-IT** | **ISTAT** | **Pros-IT** | **ISTAT** | **Pros-IT** | **ISTAT** |
| **Sample Size (n)** | 34 | 4744 | 141 | 4130 | 524 | 3409 | 156 | 2008 |
| **PCS** | | | | | | | | |
| 25°percentile | 54.3 | 50.4 | 50.8 | 45.1 | 48.9 | 37.9 | 47.2 | 30.1 |
| 50° percentile | 55.3 | 54.8 | 54.0 | 53.1 | 52.7 | 48.4 | 51.6 | 41.3 |
| 75° percentile | 55.9 | 56.0 | 55.5 | 55.5 | 55.0 | 54.3 | 54.5 | 50.4 |
| **MCS** | | | | | | | | |
| 25°percentile | 51 | 47.8 | 48.4 | 46.9 | 48.4 | 37.9 | 48.4 | 30.1 |
| 50° percentile | 52.8 | 52.8 | 52.7 | 52.8 | 52.4 | 48.4 | 52.4 | 41.3 |
| 75° percentile | 55.4 | 57.1 | 55.1 | 56.9 | 55.5 | 54.3 | 55.5 | 50.4 |

*Comparison with cancer patients*

**Table S6.** Mean and standard deviation among oncologic Italian citizens (ISTAT) and among Pros-IT patients in trajectory groups identified for MCS-12 and PCS-12

| | MCS-12 | | | PCS-12 | | |
|---|---|---|---|---|---|---|
| **Groups** | **n** | **Mean±SD** | **p-value** | **n** | **Mean±SD** | **p-value** |
| ISTAT | 598 | 41.6±12.8 | | 598 | 37.9±11.4 | |
| Trajectory 1 | 89 | 41.4±4.3 | 0.88 | 103 | 47.2±6.7 | <0.0001 |
| Trajectory 2 | 82 | 47.7±3.1 | <0.0001 | 581 | 52.1±5.0 | <0.0001 |
| Trajectory 3 | 513 | 53.7±3.3 | <0.0001 | | | |

**Table S7.** Quartiles for MCS and PCS SF-12 distributions for Pros-IT patients in each trajectory groups identified and for oncologic Italian citizens (ISTAT)

| | Pros-IT | | | |
|---|---|---|---|---|
| **MCS** | **Trajectory 3** | **Trajectory 2** | **Trajectory 1** | **ISTAT** |
| Sample Size (n) | 505 | 81 | 94 | 598 |
| 25°percentile | 51.5 | 45.8 | 39.2 | 31.3 |
| 50° percentile | 54.0 | 47.8 | 42.8 | 41.6 |
| 75° percentile | 56.2 | 50.1 | 44.5 | 53.5 |
| **PCS** | **Trajectory 2** | **Trajectory 1** | | **ISTAT** |
| Sample Size (n) | 590 | 90 | | 598 |
| 25°percentile | 49.4 | 42.8 | | 27.9 |
| 50° percentile | 53.0 | 48.4 | | 36.4 |
| 75° percentile | 55.6 | 52.3 | | 47.7 |

*The number of comorbidities in the Pros-IT and ISTAT populations according to the PCS and MCS deciles*

**Table S8.** The number of comorbidities (mean and standard deviation (SD)) in the Pros-IT population according to the PCS and MCS deciles

| | PCS | | | | | | MCS | | | | | |
|---|---|---|---|---|---|---|---|---|---|---|---|---|
| decile | 0-20 | 21-30 | 31-40 | 41-50 | 51-60 | >61 | 0-20 | 21-30 | 31-40 | 41-50 | 51-60 | >61 |
| n | 1 | 15 | 89 | 185 | 788 | 60 | 2 | 58 | 141 | 279 | 533 | 125 |
| mean | 5.0 | 2.5 | 2.0 | 1.5 | 0.9 | 0.7 | 2.5 | 1.5 | 1.6 | 1.1 | 1.0 | 0.9 |
| SD | 0.0 | 1.6 | 1.6 | 1.5 | 1.1 | 0.9 | 2.1 | 1.6 | 1.4 | 1.3 | 1.2 | 1.2 |

**Table S9.** The number of comorbidities (mean and standard deviation (SD)) in the ISTAT population according to the PCS and MCS deciles

| | PCS | | | | | | MCS | | | | | |
|---|---|---|---|---|---|---|---|---|---|---|---|---|
| decile | 0-10 | 10-20 | 20-30 | 30-40 | 40-50 | 60+ | 0-10 | 10-20 | 20-30 | 30-40 | 40-50 | 60+ |
| n | 545 | 3749 | 6524 | 10947 | 39030 | 909 | 823 | 3524 | 6383 | 13534 | 35725 | 1445 |
| mean | 4.1 | 3.9 | 2.8 | 1.8 | 0.6 | 0.9 | 3.4 | 3.1 | 2.4 | 1.4 | 0.8 | 1.7 |
| SD | 2.8 | 2.8 | 2.4 | 1.8 | 1.1 | 1.2 | 3.1 | 2.8 | 2.4 | 1.8 | 1.4 | 1.9 |

**Figure S1.** The MCS (m) and PCS (p) scores of the Pros-IT patients with color density distribution.

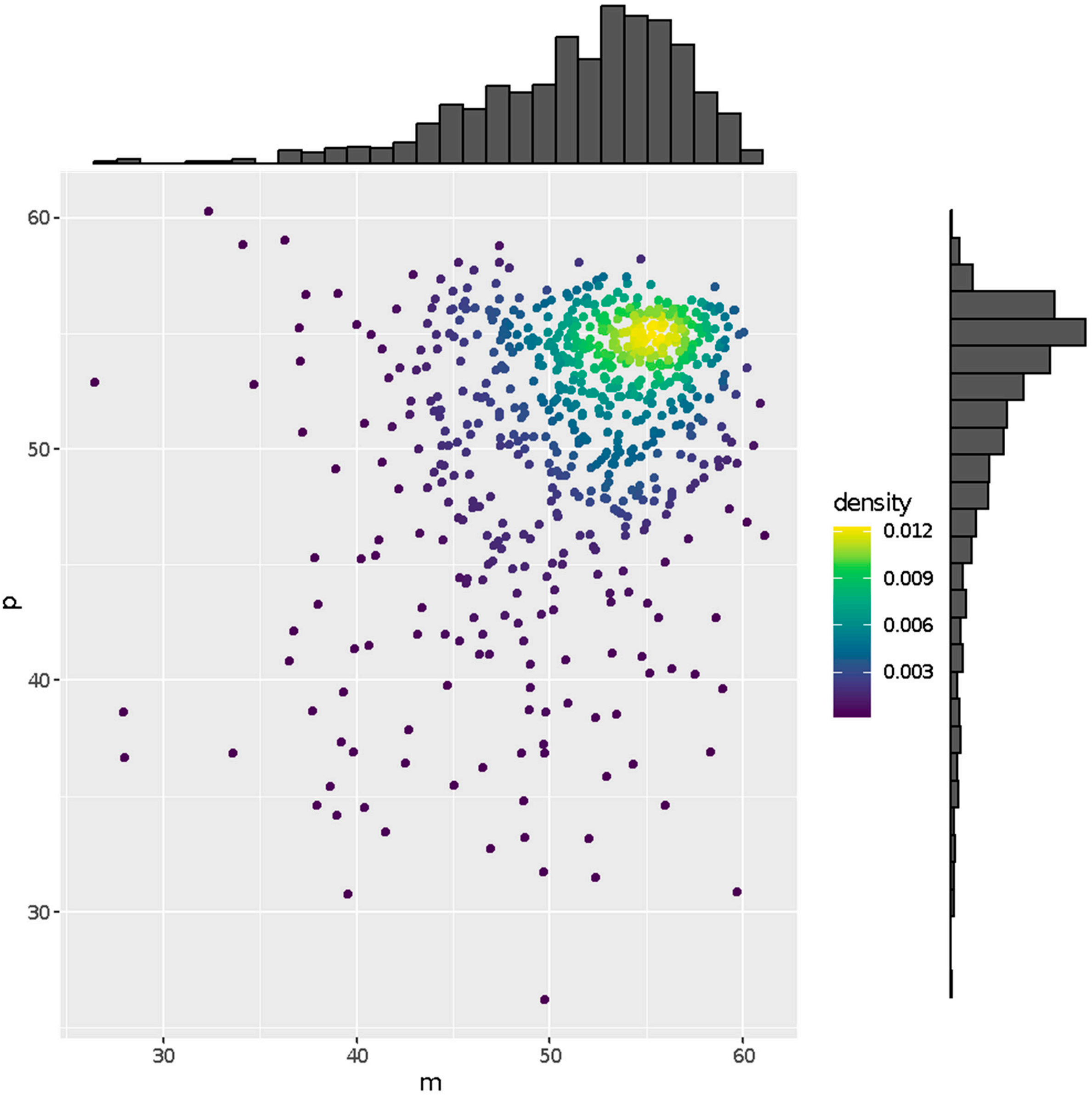

Supplement: Supplementary file 1 [file curroncol-29-00651-s001.zip › curroncol-1929770-supplementary.pdf]
